# Comparative Normal Mode Analysis of the Dynamics of DENV and ZIKV Capsids

**DOI:** 10.3389/fmolb.2016.00085

**Published:** 2016-12-27

**Authors:** Yin-Chen Hsieh, Frédéric Poitevin, Marc Delarue, Patrice Koehl

**Affiliations:** ^1^Department of Computer Science and Genome Center, University of California, DavisDavis, CA, USA; ^2^Department of Structural Biology, Stanford UniversityStanford, CA, USA; ^3^SLAC National Accelerator Laboratory, Stanford PULSE InstituteMenlo Park, CA, USA; ^4^Unit of Structural Dynamics of Macromolecules, UMR 3528 du Centre National de la Recherche Scientifique, Institut PasteurParis, France

**Keywords:** proteins, normal modes, elastic network models, viruses, Dengue, Zika

## Abstract

Key steps in the life cycle of a virus, such as the fusion event as the virus infects a host cell and its maturation process, relate to an intricate interplay between the structure and the dynamics of its constituent proteins, especially those that define its capsid, much akin to an envelope that protects its genomic material. We present a comprehensive, comparative analysis of such interplay for the capsids of two viruses from the *flaviviridae* family, Dengue (DENV) and Zika (ZIKV). We use for that purpose our own software suite, DD-NMA, which is based on normal mode analysis. We describe the elements of DD-NMA that are relevant to the analysis of large systems, such as virus capsids. In particular, we introduce our implementation of simplified elastic networks and justify their parametrization. Using DD-NMA, we illustrate the importance of packing interactions within the virus capsids on the dynamics of the E proteins of DENV and ZIKV. We identify differences between the computed atomic fluctuations of the E proteins in DENV and ZIKV and relate those differences to changes observed in their high resolution structures. We conclude with a discussion on additional analyses that are needed to fully characterize the dynamics of the two viruses.

## 1. Introduction

A major goal of molecular biology is to understand at the atomic level the functions of macromolecules and/or biological nano-machines, which are believed to be intimately related to the dynamics of their three-dimensional structures and especially their collective degrees of freedom (Koehl, 2014; Bahar et al., 2015). Our current understanding of the dynamics of macromolecules is, however, largely incomplete. This arises because only a few experimental techniques are capable of collecting time-resolved structural data, and those that can collect those data are usually limited to a narrow time window (Fromme, 2015). Similarly, state-of-the-art computational methods are limited in scope (usually in the microsecond time-scale), because of limitations in computing power (Fengand et al., 2015).

An alternate and promising approach to molecular dynamics is to infer dynamics from static structures corresponding to locally stable states (Mahajan and Sanejouand, 2015), together with reliable coarse-graining approaches to bridge the time-scale gap (Saunders and Voth, 2013; López-Blanco and Chacón, 2016). Cartesian Normal Modes, for example, represent a class of movements around a local energy minimum that are both straightforward to calculate and biologically relevant (Noguti and Go, 1982; Brooks et al., 1983; Levitt et al., 1985). The low-frequency part of the spectrum of normal modes is often associated with functional transitions, for instance, between two known states of the same macromolecule such as its apo (ligand-free) or holo (bound) form. The Elastic Network Model (ENM), introduced by Tirion in 1996, offers a particularly simple and efficient way to calculate these modes, allowing fast access to the collective dynamics of large complexes with no minimization issues as it enforces that the crystal structure is already at the energy minimum (Tirion, 1996). This model was later expanded as the Gaussian Network Model (Bahar et al., 1997) and the Anisotropic Network Model (Hinsen, 1998; Tama et al., 2000; Atilgan et al., 2001), which were shown to describe conformational changes remarkably well (Tama and Sanejouand, 2001; Delarue and Sanejouand, 2002; Zheng and Doniach, 2003; Mahajan and Sanejouand, 2015).

During the past few years, several web-servers performing on-line Normal Mode Analysis (NMA) have been set up and described: ElNemo (Suhre and Sanejouand, 2004), ENCoM (Frappier et al., 2015), Webnm@ (Tiwari et al., 2014), ANM 2.0 (Eyal et al., 2015), AD-ENM (Zheng and Doniach, 2003), NMSim (Kruger et al., 2012). We have extended and updated our own server, NOMAD-REF (Lindahl et al., 2006), with a new and user-friendlier interface, including a better visual representation of the results while at the same time enlarging the performances of the core calculation of Normal Modes in the framework of the ENM representation. New features include (i) a wider array of coarse-graining levels prior to the actual building of the ENM, and (ii) variants of the ENM that are based on a cutoff-free Delaunay tessellation of the set of atoms of the molecule of interest. With these features we depart from the original Elastic Network Model (Tirion, 1996), but keep most of its salient features, as the construction of the original Tirion Elastic Model remains available. We found for example that the Elastic Network coming from a Delaunay tessellation correctly handles PDB models with isolated domains and/or dangling ends (Xia et al., 2014). In addition, the performance of the calculation of Normal Modes has been improved to a point where it can deal with 100,000 atoms routinely, making it possible, for instance, to deal with entire virus capsids without having to resort to a symmetry-specific implementation (Simonson and Perahia, 1992; van Vlijmen and Karplus, 2005; Peeters and Taormina, 2009).

In the present paper, we show an application of some of the tools implemented in DD-NMA, the updated version of NOMAD-REF, to study the dynamics of viruses of the *flaviviridae* family, namely of Dengue virus and Zika virus.

Dengue virus (DENV) is a positive-sense RNA virus responsible for dengue fever, a tropical infectious disease whose incidence has increased drastically over the last decades, for which no prophylactic treatments are known (with the exception of eliminating the vector, i.e., mosquitoes). Today, about 3.9 billion people, or 50% of the world's population, live in areas where there is a risk of dengue transmission (Brady et al., 2012). Dengue is endemic in at least 128 countries in Asia, the Pacific, the Americas, Africa, and the Caribbean (Brady et al., 2012). The World Health Organization (WHO) estimates that close to 390 million infections occur yearly, of which 96 million manifest clinically (Bhatt et al., 2013). DENV is recognized as a potential threat to public health in the USA (Morens and Fauci, 2008). Of similar concerns are the recent outbreaks of ZIKA virus (ZIKV), another *flaviviridae* virus similar to DENV. The current ZIKV epidemic in the Americas is linked to a sudden increase in the reported cases of congenital microcephaly and Guillain Barré syndrome. This led the World Health Organization (WHO) in February 2016 to declare a “public health emergency of international concern" (WHO, 2016). As no treatments currently exist for the consequences of infections with those two viruses, and as their incidence is only expected to increase, basic research on their infection mechanisms becomes highly significant.

*Flaviviridae* genomes encode for ten different proteins, three structural proteins that form the virus particle, and seven non-structural (NS) proteins that are involved in its replication (for recent review see Meng et al., 2015). Structures of all four serotypes of DENV (Perera and Kuhn, 2008 and references therein; Zhang et al., 2012; Kostyuchenko et al., 2013, 2014; Fibriansah et al., 2015) and recently two structures of the same ZIKV strain have been published (Kostyuchenko et al., 2016; Sirohi et al., 2016). Those structures show the same global architecture, with their capsids having icosahedral symmetry consisting of 60 units, with each unit containing three copies of the E protein and three copies of protein M. The E protein is known to play a central role in many parts of the virus life cycle (Perera and Kuhn, 2008). A perhaps surprising idea that has emerged from years of studies of viruses is that their biology is deeply encoded in the dynamics of these proteins. Significant structural dynamics has been shown to occur during infection cycles, both at the level of individual proteins and at the quaternary structure level of the viral particle. These dynamics can be blocked by antibody binding (Lok et al., 2008; Teoh et al., 2012; Fibriansah et al., 2015). In addition, while the overall geometry of the viral capsid is identical in all those viruses and only small differences are observed at a finer structural scale, significant differences in stability are observed between those viruses. For example, while infection with DENV is significantly affected by temperature, infection with ZIKV remains constant at even relatively high temperatures (Kostyuchenko et al., 2016). To better understand differences between those two viruses, we investigate the dynamics of their capsid E proteins. We study those proteins independently, as well as the impact of packing imposed by the icosahedral arrangement of the virus capsid. We explore whether the differences observed, if any, are consistent with the differences observed between the structures of the capsids of DENV and ZIKV and their biological activities.

The paper is organized as follows. In the next section, we describe normal mode analysis (NMA) in the context of the Elastic Network Model. We provide an overview of the theory and discuss the different options for choosing its parameters, namely the choice of coarse-graining level, the choice of the elastic force constants, and the cutoff for selecting the pairs of atoms that belong to the ENM. In the following section, we provide a description of the algorithms used to implement NMA within our new server DD-NMA, with a special focus on scalability to large molecular systems. In the Results section, we discuss the applications of DD-NMA to study the dynamics of DENV and ZIKV, focusing on the differences and similarities of the dynamics of their capsid E protein. We conclude the paper with a brief discussion on future developments of normal mode analysis applied to viral structures.

## 2. Normal mode analysis

### 2.1. Normal mode analysis based on the tirion elastic network model

The Elastic Network Model (ENM) was originally introduced by Tirion (1996). It is a model that captures the geometry of the molecule of interest in the form of a network of inter-atomic connections, linked together with elastic springs. Two categories of normal mode analyses based on ENMs are widely used today, namely, the Gaussian Network Model (GNM) (Bahar et al., 1997; Haliloglu et al., 1997) and the anisotropic network model (ANM) (Tirion, 1996; Atilgan et al., 2001). Here we follow the latter model, in which the energy of the molecule is equated to the harmonic energy associated with these springs. This defines a quadratic energy on the inter-atomic distances. Let *M* be a biomolecule containing *N* atoms, with atom *i* characterized by its position *X*_*i*_ = (*x*_*i*_, *y*_*i*_, *z*_*i*_). The whole molecule is then described by a 3*N* position vector *X*. For two atoms *i* and *j* of *M*, we set *r*_*ij*_ = |*X*_*i*_ − *X*_*j*_| and $r_{ij}^{0}=|X_{i}^{0}-X_{j}^{0}|$ to be their Euclidean distances in any conformation *X* and in the ground-state conformation *X*^0^ (usually the X-ray structure), respectively. The total potential *V*_*ENM*_ of the biomolecule is then set to:

(1) $$V_{ENM}(X)=\frac{1}{2}\sum_{i=1}^{N}\sum_{j>i}k_{ij}{\left(r_{ij}-r_{ij}^{0}\right)}^{2}\Theta (R_{c}-r_{ij}^{0})$$

where *R*_*c*_ is a cutoff distance, *k*_*ij*_ is the force constant of the “spring" formed by the pair of atoms *i* and *j*, and Θ(*x*) is the Heaviside unit step function, i.e., Θ(*x*) = 0 if *x* < 0 and Θ(*x*) = 1 otherwise. Both *R*_*c*_ and *k*_*ij*_ are discussed in detail below.

In the normal mode framework, the potential *V*_*ENM*_ is then approximated with a second-order Taylor expansion in the neighborhood of the ground state *X*^0^:

(2) $$\begin{array}{l} V_{ENM}(X)\approx V_{ENM}(X^{0})+\nabla V_{ENM}{\left(X^{0}\right)}^{T}(X-X^{0})\\ \;+\frac{1}{2}{\left(X-X^{0}\right)}^{T}H(X-X^{0}) \end{array}$$

where ∇*V*_*ENM*_ and *H* are the gradient and Hessian of *V*_*ENM*_, respectively. Note that based on Equation 1, $V_{ENM}\left(X^{0}\right)=0$ and $\nabla V_{ENM}\left(X^{0}\right)$ is the null vector (i.e., *X*^0^ is a global minimum of *V*_*ENM*_ by definition). The ENM energy is then simply

(3) $$V_{ENM}(X)\approx \frac{1}{2}{\left(X-X^{0}\right)}^{T}H(X-X^{0})$$

The 3 × 3 submatrix *Hij* of the Hessian *H* corresponding to two atoms *i* and *j* that are in contact is given by:

(4) $$\begin{array}{l} Hij=-\frac{k_{ij}}{{\left(r_{ij}^{0}\right)}^{2}}(X_{i}-X_{j}){\left(X_{i}-X_{j}\right)}^{T}\\ \;=-\frac{k_{ij}}{{\left(r_{ij}^{0}\right)}^{2}}\left[\begin{array}{ccc} {\left(x_{i}-x_{j}\right)}^{2} & \left(x_{i}-x_{j})(y_{i}-y_{j}\right) & \left(x_{i}-x_{j})(z_{i}-z_{j}\right)\\ \left(y_{i}-y_{j})(x_{i}-x_{j}\right) & {\left(y_{i}-y_{j}\right)}^{2} & \left(y_{i}-y_{j})(z_{i}-z_{j}\right)\\ \left(z_{i}-z_{j})(x_{i}-x_{j}\right) & \left(z_{i}-z_{j})(y_{i}-y_{j}\right) & {\left(z_{i}-z_{j}\right)}^{2} \end{array}\right] \end{array}$$

and the 3 × 3 submatrix *Hii* on the diagonal of *H* is then given by:

(5) $$Hii=-\sum_{j=1,N}Hij$$

In Cartesian coordinates, the equations of motion defined by the potential *V*_*ENM*_ are derived from Newton's equation:

(6) $$\frac{d^{2}X}{dt^{2}}=-H(X-X^{0})$$

Writing the solution to this equation as a linear sum of intrinsic motions (the “normal modes" of the system),

(7) $$X_{j}=\sum_{k=k_{0}}^{3N}A_{jk}\alpha_{k}cos(\omega_{k}t+\delta_{k})$$

we get a standard eigenvalue problem,

(8) HA=MAΩ

The eigenfrequencies ω are given by the elements of the diagonal matrix Ω, namely $\omega_{i}^{2}=\Omega \left(i,i\right)$. The eigenvectors are the columns of the matrix *A*, and the amplitudes and phases, α_*k*_ and δ_*k*_, are determined by initial conditions. The matrix *M* is a diagonal matrix containing the masses of the atoms. We note that the first six eigenvalues in Ω are equal to 0, as they correspond to global translations and rotations of the biomolecule. To characterize the internal motions of the biomolecule, the sum in Equation 8 runs then from *k*_0_ = 7 up to 3*N*, the number of degrees of freedom of the system.

### 2.2. Parametrization: choosing the representation of the molecule

The first requirement when building an ENM is to define the set of atoms on which it is based. Although all atoms could be used, it appears natural to lower the dimensionality of the system, namely “coarse-graining,” when large biomolecules are considered, or in the context of a harmonic approximation to its energy as is the case in ENM (Tozzini, 2005). Coarse-grained models have long been used for studying protein folding and aggregation. They enable the exploration of large length scales and time scales that are usually inaccessible to all-atom models in explicit solvent (Saunders and Voth, 2013; Kmiecik et al., 2016). Combined with enhanced configuration search methods, these simplified models with various levels of granularity offer the possibility to determine equilibrium structures and to compare folding kinetics and thermodynamics quantities with the corresponding values obtained by experimental techniques. In their pioneer work from 1976, Levitt and Warshel (1976) developed the foundation of coarse-graining for protein folding. They were able to fold the 58-residue BPTI protein within 6.5 Å from its experimental structure using a two-bead representation for each residue in the protein. This representation included the Cα and the centroid of the side chain to define a residue. They used an effective implicit solvent force field such that the atoms of the solvent need not be considered explicitly, and successive minimizations and normal mode thermalization to fold BPTI. Since then, various levels of granularity have been developed, from lattice representations to multi-bead representations, and from single atom to multiple-atom residue-level representations (Kmiecik et al., 2016). The positions of those beads are either defined by known atoms (usually the Cα), or by fitting to capture the dynamics of the full molecular systems (Zhang et al., 2008, 2011; Li et al., 2016). For all the analyses of virus structures considered in this paper, we used the Cα-only representation.

### 2.3. Parametrization: choosing the spring force constants

In the original ENM introduced by Tirion, the elastic constants *k*_*ij*_ are set to be the same for all pairs of atoms. In other models, *k*_*ij*_ vary for different pairs of atoms. For example, Ming and Wall (2005) employed an enhanced ENM in which the interactions of neighboring Cα atoms on the backbone were strengthened to reproduce the correct bimodal distribution of density-of-states from an all-atom model. Kondrashov et al. (2006) used a strategy in which they classified residue interactions into several categories corresponding to different physical properties. The elastic constants can also be adjusted to have the fluctuations of the atoms of the molecule computed from the equations of motions given by Equation (7) to match the atomic fluctuations captured experimentally and usually reported as B-factors. Many methods have been developed for that purpose (see for example Xia et al., 2013, 2014 and references therein). Among those methods, the one proposed by Erman (2006) is worth discussing. Erman developed an iterative algorithm to update the Kirchhoff matrix of a Gaussian Network Model, in which the connections of neighboring Cα atoms on the backbone of the protein of interest are fixed, and the strengths of the interactions between pairs of residues are varied until a good fit between experimental B-factors and computed fluctuations is obtained. While this approach generates a really good fit between those two representations of fluctuations, a significant number of the optimized spring force constants are found to be negative. While such negative values are not forbidden, they do hint at the possibility of overfitting. This is in accordance with (Fuglebakk et al., 2013), who recently suggested that such a refinement procedure leads to overfitting, and not to a better dynamic model for the molecule. As such, in this study we assign the same value for all *k*_*ij*_, following the initial ENM of Tirion (1996).

### 2.4. Parametrization: the cutoff parameter *R_c_*

In standard implementations, the cutoff distance *R*_*c*_ and the force constant *k* are set constant for all pairs of residues. Their values, however, differ between the two models. For example, the cutoff distance *R*_*c*_ for GNM is usually set in the range of 7 to 8 Å (Kundu et al., 2002) while in ANM larger values are usually considered in the range from 13 to 15 Å (Eyal et al., 2006). There are, however, no guidelines as to which values are best and sometimes different implementations lead to contradicting optimal values. To circumvent these discrepancies, several authors have proposed to include all pairs of residues in a protein and to assign different force constants to their corresponding springs that relate to their lengths at rest (see for example Hinsen, 1998; Kovacs et al., 2004; Yang et al., 2009). In these methods, the use of a plain cutoff distance is avoided. The number of pairs of atoms considered, however, is large and scales as *N*^2^, where *N* is either the total number of atoms in the biomolecule considered, or its number of residues. Such a quadratic behavior makes these methods unfit for studying large systems. To study the capsids of DENV and ZIKA, we have considered a traditional cutoff ENM, with the cutoff set to 14 Å, unless specifically noted.

## 3. Materials and methods

We have used our own software package, DD-NMA, to perform all the analyses discussed in the Results section. In the following, we highlight some of the elements of DD-NMA that are relevant to the analysis of large systems. We note that DD-NMA is available as a web-based service at http://lorentz.dynstr.pasteur.fr/suny/index.php?id0=delaunaynma#welcome.

### 3.1. An efficient algorithm to diagonalize the hessian of the elastic potential *V_ENM_*

The Hessian matrix of *V*_*ENM*_ is a 3*N* × 3*N* symmetric, real-valued matrix whose elements are described by Equation (4). The theory described above calls for diagonalizing this matrix, as its eigenvalues and eigenvectors provide the frequencies and directions, respectively, of the normal modes of the molecular systems under study. While many methods exist for solving such an eigenvalue problem, see Golub and van der Vorst (2000), many of those methods break down when *N* becomes large, both in terms of computing time and memory requirements. The Hessian matrix is highly sparse as only a subset of all atom pairs are usually considered (see previous section for a discussion of this point), but this is not enough to offset the computing requirements as the matrix *A* of eigenvectors is usually not sparse. However, in her original paper, Tirion (1996) had recognized that the lowest frequency normal modes can capture most of the dynamics of the protein of interest. This observation has since been supported by further evidence that the lowest-frequency normal modes generated from ENM conform with conformational changes observed by X-ray and NMR experiments (Kim et al., 2002; Maragakis and Karplus, 2005; Kurkcuoglu et al., 2009) as well as with the results of MD simulations (Rueda et al., 2007; Orellana et al., 2010; Leioatts et al., 2012). While it is unclear as to how many of those low frequency normal modes need to be considered (Petrone and Pande, 2006), it remains that only a small fraction of the eigenvalues and eigenvectors of the Hessian matrix need to be computed, which leads to the opportunity to use powerful iterative algorithms for computing those quantities. The most successful family of such algorithms is based on the efficient Krylov subspace method, as it allows for targeting only a subset of the eigenvalue spectrum of a matrix. We provide below the rationale behind this method to compute the eigenvalues with lowest magnitude of the Hessian matrix.

An intuitive method for finding the largest eigenvalue of a given *N* × *N* matrix A is the power iteration. Starting with an initial random vector *x*, this method calculates *Ax*, *A*^2^*x*, *A*^3^*x*,… iteratively, storing and normalizing the result into *x* at every iteration. The corresponding sequence of Rayleigh quotient *R*_*i*_

(9) $$R_{i}=\frac{x^{T}A^{i}x}{x^{T}x}$$

converges to the largest eigenvalue of A, while *x* itself converges to the corresponding eigenvector. However, much potentially useful computation is wasted by using only the final result. This suggests that, instead, the so-called Krylov matrix is to be formed:

(10) $$K_{n}=\left[\begin{array}{ccccc} x & Ax & A^{2}x & \ldots & A^{n-1}x \end{array}\right]$$

The columns of this matrix are not orthogonal, but an orthogonal basis can be constructed via a stabilized Gram–Schmidt orthogonalization. The resulting vectors are a basis of the Krylov subspace, $K_{n}$. The vectors of this basis give good approximations of the eigenvectors corresponding to the *n* largest eigenvalues of *A*. In a similar manner, the smallest eigenvalues of *A* can be computed by applying this strategy to either *A*^−1^, or by applying a spectral shift, i.e., by computing the largest eigenvalues of *A* − λ_*max*_*I*, where λ_*max*_ is the largest eigenvalue of *A*.

We use the ARPACK implementation of a variant of this approach, the implicitly restarted Arnoldi iteration method (Lehoucq et al., 1998).

### 3.2. Atomic fluctuations computed from normal modes

From the normal modes of the ENM, it is possible to compute the mean square fluctuations of the positions of the atoms according to:

(11) $$<\Delta X_{i}^{2}>=\frac{k_{B}T}{m_{i}}\sum_{k=7}^{m}\frac{A_{ik}^{2}}{\omega_{k}^{2}}$$

where Δ**X**_*i*_ and *m*_*i*_ are the displacement vector and mass of vertex *i*, respectively, *k*_*B*_ is the Boltzmann's constant, *T* is the temperature considered, *A*_*ij*_ is the *i*-th component of the *j* eigenvector *A*_*j*_ of the Hessian, and ω_*i*_ is its associated eigenvalue. The summation should run over all the modes of the system (excluding the six modes for rigid body transformations); it is truncated here to the first *m* = 100 modes, as those low frequency modes are usually responsible of most of the atomic fluctuations (see above).

### 3.3. Correlated motions within a biomolecule

The Boltzmann distribution for the approximate potential of the ENM (see Equation 3) is described by a multivariate Gaussian distribution with a covariance matrix proportional to the inverse of the Hessian *H*. Because of the six rigid motions captured by the six normal modes with 0 frequencies, the inverse of *H* is in fact not properly defined. We can, however, compute a pseudo-inverse by ignoring those zero energy modes; this pseudo-inverse can be regarded as a covariance matrix of internal deformation:

(12) $$C=\sum_{k=7}^{M}\frac{1}{\omega_{k}^{2}}A_{k}A_{k}^{T}$$

where ω_*k*_ and *A*_*k*_ are the *k* − *th* eigenvalues and eigenvectors, respectively. Note that *C* is a 3*N* × 3*N* matrix. The summation extends from *k* = 7, the first non-zero mode, to *M*, the highest mode considered (up to 3*N*). To obtain a scalar quantification of the correlation of the motions of two atoms *i* and *j*, a correlation matrix *P* is computed, following Ichiye and Karplus (1991):

(13) $$P_{ij}=\frac{tr(C_{ij})}{\sqrt{tr(C_{ii})tr(C_{jj})}}$$

The values *P*_*ij*_ range from −1 to +1, with a negative correlation value indicating an anticorrelated motion, and a positive correlation value identifying a correlated pattern of dynamics between the two atoms considered. These values are stored into a cross-correlation matrices CCM that is used to visualize correlations of motion within the molecule under study.

## 4. Results and discussion

**Figure 1:**
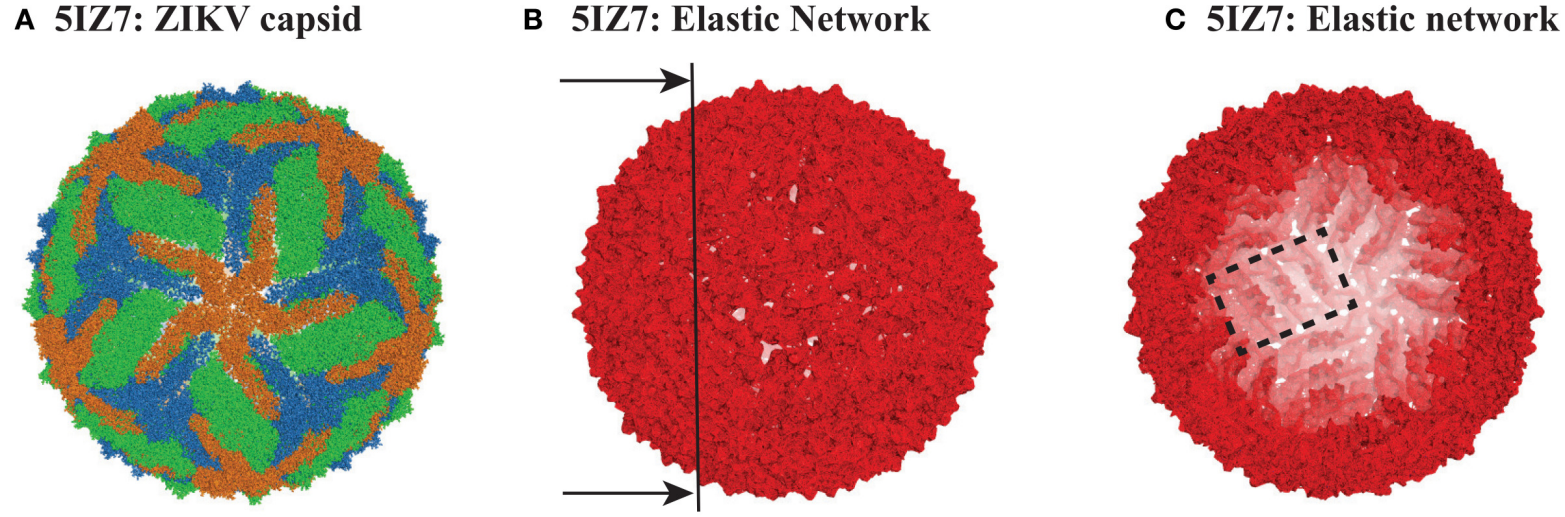
**The capsid of ZIKV. (A)** Cartoon representation of the capsid of ZIKV (PDB file 5IZ7). The capsid includes 180 copies of protein E. The three E proteins from each asymmetric unit are colored green, orange, and blue. **(B)** The elastic network of the capsid of ZIKV, constructed from the Cα only, with a cutoff *R*_*c*_ = 14 Å. **(C)** Inside view of the elastic network, obtained by cutting the full elastic network along the plane shown as a line on **(B)**. Note that it is possible to identify rafts, as illustrated with one raft being contoured with a dashed rectangle (see text for details). All three panels were generated using Pymol (http://www.pymol.org).

DENV and ZIKV are both members of the *flaviviridae* family. DENV serotype 1 and ZIKV (which are the focus of this study) share 53% sequence identity (Kostyuchenko et al., 2016). Their particles share a common fold, with their capsids having icosahedral symmetry. Those capsids are formed of 60 asymmetrical units, with each unit containing three copies of E protein (495 and 504 residues in DENV and ZIKV, respectively) and three copies of the membrane protein M (74 and 75 residues in DENV and ZIKV, respectively). The high resolution cryo-EM structures of all four serotypes of DENV, as well as the structure of one strain of ZIKV, are available in the Protein Data Bank (Zhang et al., 2012; Kostyuchenko et al., 2013, 2014; Fibriansah et al., 2015; Kostyuchenko et al., 2016; Sirohi et al., 2016). Here we focus on the structure of the mature form of serotype 1 of DENV, with PDB code 4CCT (Kostyuchenko et al., 2013), and of the equivalent mature form of ZIKV, as given by one of the recently published structures, with PDB code 5IZ7 (Kostyuchenko et al., 2016). Those two structures were shown to be very similar, with only small differences that will be discussed in light of their dynamics. A cartoon representation of ZIKV is given in Figure 1A. The DENV capsid shows the same architecture.

The PDB file for 4CCT only contains Cα atoms. For consistency, we used Cα only representations of 4CCT and 5IZ7. We isolated from those two files all the Cα atoms of the viral capsid. For both viruses, we considered E protein in four different environments: isolated, MONO, (corresponding to chain A in the asymmetric unit of 4CCT and chain B of the asymmetric unit of 5IZ7), within the asymmetric unit, UNIT, within a raft, RAFT, and within the whole capsid structure, FULL. The corresponding complexes MONO, UNIT, RAFT, and FULL contain 495, 1707, 3414, and 102420 residues for 4CCT, respectively, and 504, 1737, 3474, and 104220 residues for 5IZ7, respectively. We generated elastic networks for all those eight complexes using a cutoff procedure, with the cutoff set to 14 Å. Figures 1B,C illustrate the elastic network for the FULL complex for ZIKV (5IZ7). We note that this elastic network follows the surface of the capsid virus and does not include any edges that cross the interior of the capsid; this is a direct consequence of the cutoff that is used. The inside of the geometric structure formed by the elastic network reveals the presence of rafts (one such raft is shown inside a rectangle in Figure 1C), namely three dimers of E protein lying parallel to each other. Once the elastic networks were established, we computed the hundred lowest normal modes for each of them, using the procedure detailed in the Methods section.

We emphasize that the elastic networks for the full capsids were computed using the empty protein shells, following previous studies of viral particles using ENM and their normal modes (Tama and Brooks III, 2002, 2005; Kim et al., 2003; Chennubotla et al., 2005; Rader et al., 2005; Polles et al., 2013). This setting is expected to be satisfactory as the stability of the empty capsid is guaranteed by the geometric construction of the ENM, which makes up for the missing stabilizing interactions of the coat proteins and RNA. We note that the latter were not resolved in the cryo-EM structures we considered.

### 4.1. Characterizing the low frequency normal modes of DENV and ZIKV

**Figure 2:**
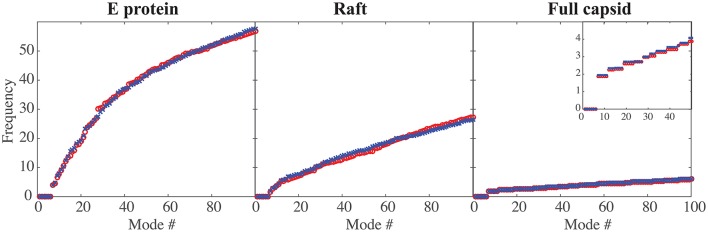
**Comparing the low frequencies of the normal modes of DENV and ZIKA**. The frequencies of the first hundred normal modes of DENV (red circle, o) and ZIKV (blue cross, x) are plotted against the normal mode index (#), for the E protein by itself (left), for a raft (middle), and for the full capsid (right). The frequencies are in arbitrary units, as the force constants are also in arbitrary units. Note the decrease in the amplitude of those frequencies as the size of the complex increases. The insert in the right panel shows an enlargement for the first 50 normal modes; it highlights the degeneracy of the normal modes for a full capsid.

In Figure 2 we compare the frequencies of the first hundred normal modes of the MONO, RAFT, and FULL complexes of DENV and ZIKV. As expected, the first six frequencies are found equal to zero, for all complexes considered, as those frequencies correspond to the rigid motions (three translations and three rotations). The larger the protein complex, the more the spectra of frequencies of the normal modes are shifted toward lower frequencies, indicating the presence of more collective motions in protein oligomers. The spectra of frequencies for the full capsids reveal the presence of degeneracy, namely repeating frequencies, that correspond to symmetries in the capsid. All the differences observed in the three complexes are conserved between DENV and ZIKV. We note also the nearly perfect match between the normal mode frequency spectra of the two viruses.

### 4.2. Correlated dynamics of E proteins in the capsids of DENV and ZIKV

**Figure 3:**
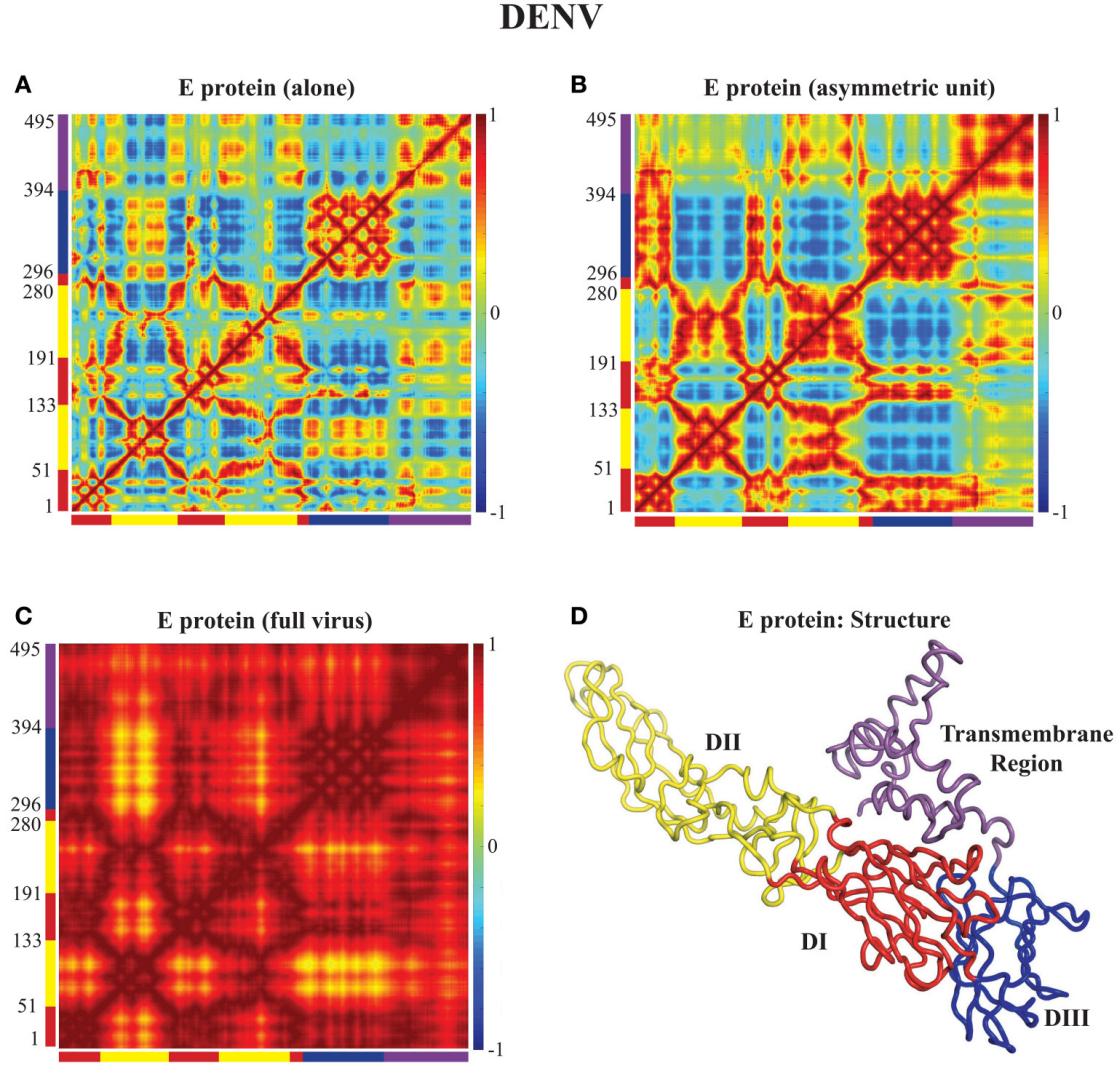
**Correlated motions in the DENV E protein**. Cross Correlation Matrices (CCM) obtained from the 94 first non-zero modes for the E protein alone (MONO, **A**), the E protein in the asymmetric unit (UNIT, **B**), and the E protein in the whole capsid (FULL, **C**). Those plot show correlations between the motions of Cα atoms in each complex considered. Both axes of a matrix are the amino acid residue index. Each cell in a matrix shows the correlation between the motions of two residues (Cα atoms) in the protein on a range from −1 (anticorrelated, blue) to 1 (correlated, red), with 0 conferring no correlation. **(D)** The E protein is shown in cartoon mode. The color code for the structure in **(C)** as well as for the X and Y axes of the CCM plots in **(A)** to follows the standard designation of the E protein domains I (red), II (yellow), and III (blue). The transmembrane domain is shown in purple. Panel **(D)** was generated using Pymol.

**Figure 4:**
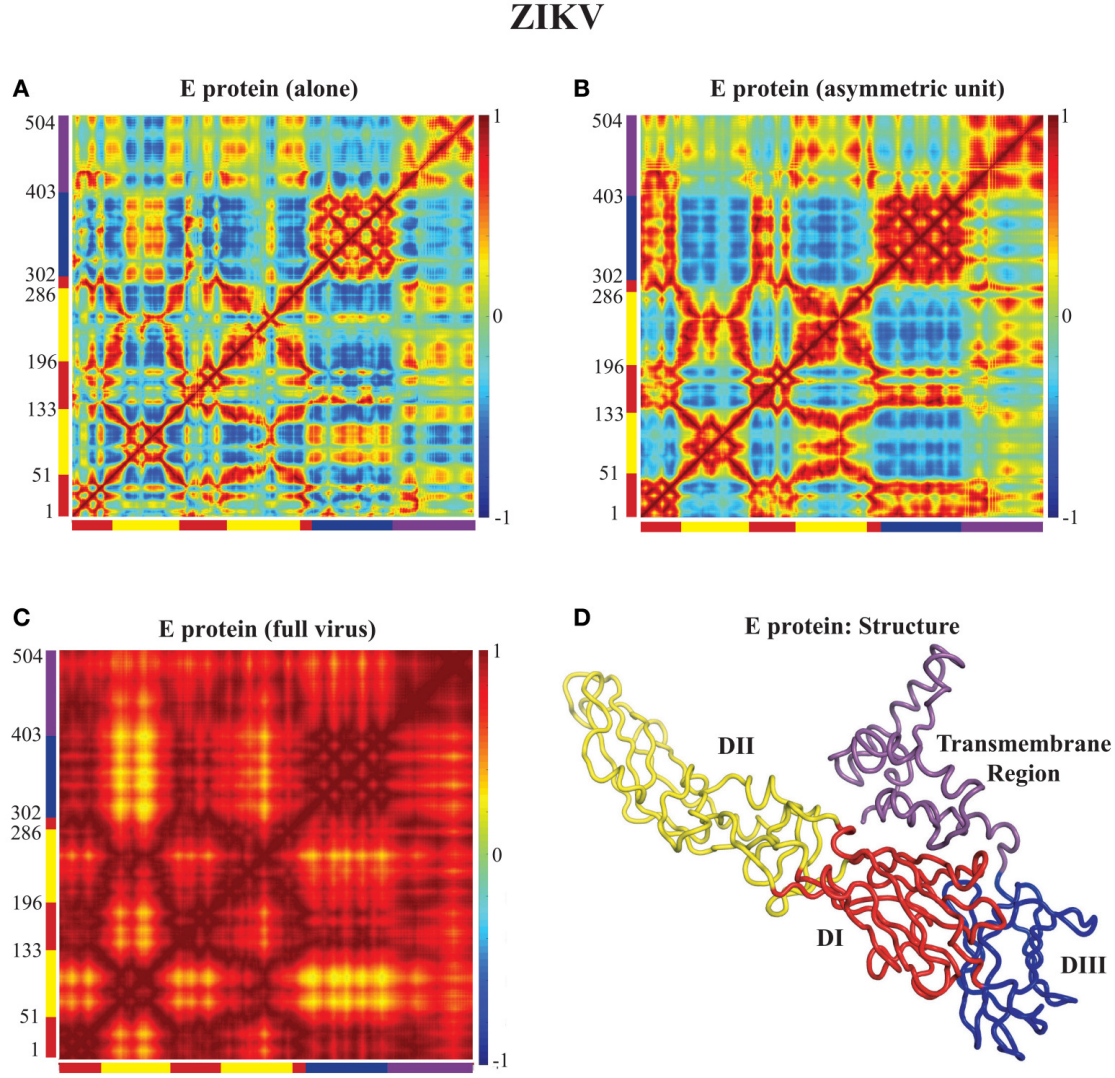
**Correlated motions in the ZIKV E protein**. Cross Correlation Matrices (CCM) obtained from the 94 first non-zero modes for the E protein alone (MONO, **A**), the E protein in the asymmetric unit (UNIT, **B**), and the E protein in the whole capsid (FULL, **C**). **(D)** The E protein is shown in cartoon mode. Colors and layout follow the same schemes as in Figure 3.

In Figures 3, 4 we assess the extent to which packing influences the dynamics of the E protein of DENV and ZIKV, respectively. For both viruses, the cross correlation matrices (CCM) for E protein vary significantly between the MONO, UNIT, and FULL complexes. The CCM for the E protein alone reveals significant positive correlations within each of the three domains I, II, and III. Domains II and III exhibit both positive and negative correlations in their atomic fluctuations, while the motions of domain I are only weakly correlated to the motions of domain II and III. When the dynamics of the E protein are studied in the context of the asymmetric unit, the same positive correlations are observed within each of the three domains. The interactions between the domains change significantly, however. In the UNIT complex, the dynamics of domain II are strongly anticorrelated to the dynamics of domain III, while domain I is correlated positively with domain III. In the full viral capsid, the internal dynamics of the E protein remain mostly as observed in the asymmetric unit. The only difference is the addition of a global positive correlation over the full protein that comes from concerted dynamics within the capsid. In all three oligomeric states, the transmembrane domain shows weak positive correlation with domain II.

All the differences in dynamics observed between isolated E proteins and E proteins in the whole capsid are conserved between DENV and ZIKV.

### 4.3. Correlated dynamics of rafts of E proteins in the capsids of DENV and ZIKV

**Figure 5:**
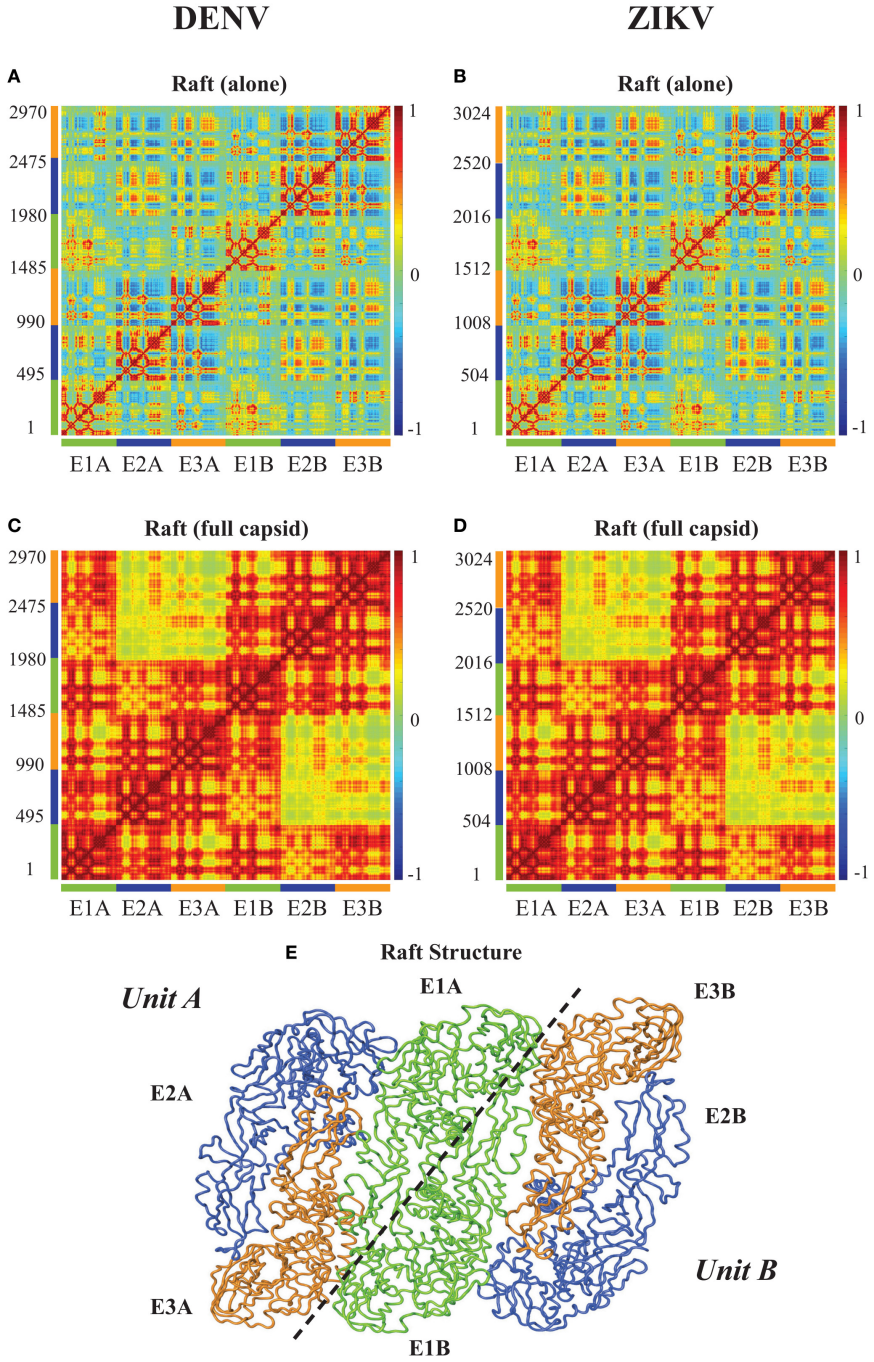
**Correlated motions in the a E protein raft**. Cross Correlation Matrices (CCM) obtained from the 94 first non-zero modes for a E protein raft alone (UNIT), and a raft in the whole capsid (FULL) for DENV **(A,C)**, and for ZIKV **(B,D)**. X axes and Y axes are residue indices. The positions of the six E proteins are marked, with labels and color codes defined on the structure in **(E)**. **(E)** Cartoon model for the raft. Note that a raft includes two asymmetric units, labeled Unit A and Unit B. The first E protein of each unit, E1A and E1B form a dimer. Panel **(E)** was generated using Pymol.

Figures 3, 4 reveal the effects of packing in the viral capsid on the dynamics of one E protein. We performed a similar analysis on a larger structure of the capsid, namely a raft. A raft is formed from six E proteins forming 3 dimers arranged in a parallel manner, resulting from the combination of two asymmetrical units (see Figure 5E). The whole capsid contains 30 such rafts. In Figures 5A–C, we assess the extent to which packing influences the dynamics of such rafts for both DENV and ZIKV. In the CCM for the raft alone (Figures 5A,B for DENV and ZIKV, respectively) we clearly identify the six E proteins along the diagonal. Each of those E proteins exhibits dynamics correlation patterns equivalent to those observed in the E protein when it is in the asymmetrical unit. The interactions between the E proteins are consistent with the structure of the raft. The first E proteins of the two asymmetrical units, proteins E1A and E1B, show strong positively correlated dynamics. Those two proteins form a dimer in the raft. In contrast, proteins E2A and E3A in Unit A, and proteins E2B and E3B in Unit B have a pattern of interactions that include both positively correlated and negatively correlated motions, depending on their domains: for example, domains III have negative correlations between the two proteins, while domains II are positively correlated between the two proteins. The pair of proteins (E2A, E3A) shows weak correlated dynamics with the pair of proteins (E2B, E3B), with a chessboard pattern (i.e., positive correlations between E2A and E2B, and negative correlations between E3A and E3B).

The CCMs for a raft included in the whole capsid (Figures 5C,D for DENV and ZIKV, respectively) reveal different patterns than those described for the raft alone, highlighting again the impact of packing in the virus environment. There is a high level of positive correlation of motions within each of the units A and B. The proteins E1A and E1B that form a dimer at the center of the raft are mostly interacting with themselves in the raft alone, while they show strong levels of positive correlations with all three E proteins of the opposing unit in the raft when considered within the whole capsid. In contrast, the pairs of proteins (E2A, E3A) and (E2B, E3B) present significantly lower correlation when considered in the whole capsid compared to the raft alone. Such a behavior would favor concentration of concerted internal motions in a few E protein dimers at the center of the rafts in the whole viral capsid instead of a more uniform spread of concerted motions.

Similar to the findings for the dynamics of the E proteins, the differences in dynamics observed between isolated rafts and rafts in the whole capsid are conserved between DENV and ZIKV.

### 4.4. Atomic fluctuations within the E proteins of the capsids of DENV and ZIKV

**Figure 6:**
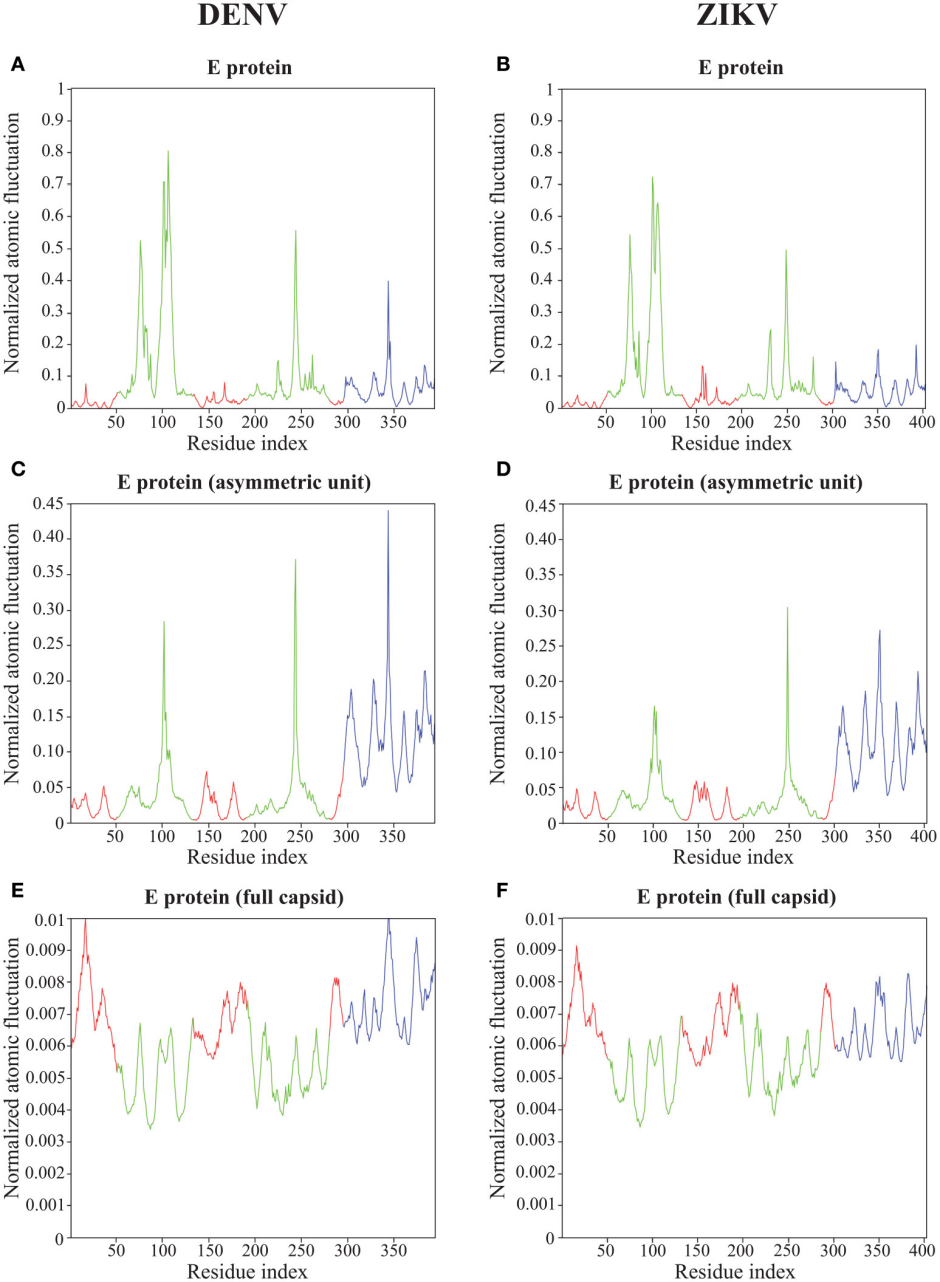
**Atomic fluctuations in the DENV and ZIKV E proteins**. The atomic displacement fluctuations obtained from the 94 first non-zero modes for the E protein alone (MONO, **A,B**), the E protein in the asymmetric unit (UNIT, **C,D**), and the E protein in the whole capsid (FULL, **E,F**) are plotted as a function of the residue number for both DENV (PDB file 4CCT) and ZIKV (PDB file 5IZ7). The Y axis represents normalized displacements (see text for details). The color code follows the standard designation of the E protein domains for domains I (red) and III (blue), while domain II has been colored green to enhance visibility.

The normalized squared atomic fluctuations for each Cα atom in the E protein of DENV and ZIKV were calculated as the sum of their displacements along the first 94 non-zero modes, weighted by the reciprocal of the eigenvalues, as given by Equation (11). For both viruses, the calculation was performed in three states for the E protein, namely the MONO, UNIT, and FULL complexes described above. The absolute values of the amplitudes of the fluctuations computed using Equation (11) are somewhat arbitrary, as they depend on the parametrization of the elastic network, namely on the cutoff values *R*_*c*_ and the strength of the force constants *k*_*ij*_. While it is possible to select those parameters such that a good fit is obtained between the computed fluctuations and experimental B-factors, we prefer not to, following the advice of Fuglebakk et al. (2013) that warned on possible overfitting problems. Instead, we just normalize the computed fluctuations for an atom *i* using:

(14) $$<\Delta_{N}X_{i}^{2}>\;=\frac{<\Delta X_{i}^{2}>-\min(<\Delta X^{2}>)}{\max(<\Delta X^{2}>)-\min(<\Delta X^{2}>)}$$

where the min and max values are computed over all Cα atoms of the molecule considered. To enable comparison, we computed the min and max values from the fluctuations observed in the E protein alone, and applied those to normalize the fluctuations of all three states considered, i.e., MONO, UNIT, and FULL. Results for DENV and ZIKV are shown in Figure 6.

Not unexpectedly, the amplitude of the atomic fluctuations within the E protein decreases as the protein is more constrained, from a (normalized) range between 0 and 1 in the E protein alone (Figures 6A,B), to a range between 0 and 0.01 in the full capsid (Figures 6E,F). Of significance is the change in dynamics observed in the kl-loop between domains I and II (the DI-DII hinge, residues 280–290) between the stand alone E protein and the capsid. In the former, this loop region is predicted to be rigid, while in the latter it is found to be significantly more dynamic. This hinge is thought to be important to flip the domain DII to expose the fusion loop during the fusion event (Modis et al., 2003; Zhang et al., 2004; Kostyuchenko et al., 2016). In contrast, the HI-loop in the putative receptor binding domain DIII (residues 230–240) is found to be more dynamic in the E protein alone than in the whole capsid. DENV and ZIKV show the same dynamical behavior in both loops (the kl- and HI-loops).

The two plots showing the atomic fluctuations computed from normal modes in the E proteins are globally similar between DENV and ZIKV in all oligomeric states (Figure 6). There are, however, some localized differences that are worth discussing. There is a putative increase in dynamics in the region 150–160 in ZIKV compared to DENV that is most marked in the E protein monomer, but still present it its oligomeric states. This region corresponds to the Glycan loop, which contains a glycosylation site (Asn154 in ZIKV and Asn153 in DENV). It was found to be the region with the biggest structural differences (up to 6 Å) in the cryo-EM structures of ZIKV and DENV (Sirohi et al., 2016). Our calculations were performed in the absence of the sugar moities on the Asparagine. We believe however that our results highlight an intrinsic difference in the dynamics of the Glycan loops of DENV and ZIKV that is worth exploring further. In contrast to the Glycan loop, the region 340–350 is found to be less dynamic in ZIKV than in DENV in all oligomeric states of their E proteins. This region corresponds to the C strand and CD loop in domain III. Based on the differences in the structures of the DENV and ZIKV capsids, Kostyuchenko et al. (2016) hypothesized that the presence of an additional amino acid in the C strand in ZIKV was responsible for a rearrangement of the structure locally that is possibly responsible for the increased thermal stability of ZIKV. Our results indeed suggest a more rigid C strand in ZIKV compared to DENV. The exact relationship between this decrease in atomic fluctuations and thermal stability is unknown.

**Figure 7:**
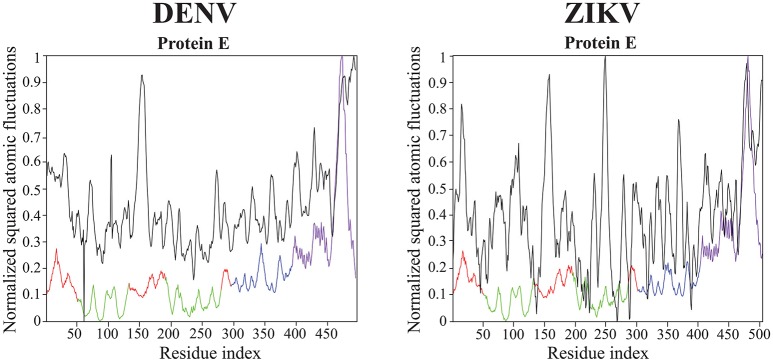
**Comparison of normalized experimental and computed atomic fluctuations in the DENV and ZIKV E proteins**. The computed atomic displacement fluctuations were obtained from the 94 first non-zero modes of the whole capsid shell. The experimental fluctuations are taken from the cryo-EM structures of DENV (4CCT, Kostyuchenko et al., 2013) and ZIKV (5IZ7, Kostyuchenko et al., 2016) The color code for the computed atomic fluctuation is: E protein domain I, red, II, green, III, blue, and transmembrane domain, purple.

All results on dynamics presented above are based on atomic fluctuations and dynamic correlations computed from normal mode analyses. In Figure 7 we compare those normalized computed atomic fluctuations for the Cα atoms of the E protein in the full capsid structure with the corresponding normalized experimental B-factors extracted from the PDB files 4CCT and 5IZ7 for DENV and ZIKV, respectively. Overall, the profiles show qualitative similarities over the full range of residues in E protein. The correlation coefficients between the experimental B-factors for DENV and ZIKV and the computed atomic fluctuations are 0.58 and 0.45, respectively. Those values are modest. We note that it would be possible to obtain significantly better correlations if the elastic constants *k*_*ij*_ assigned to the links of the networks were fitted to improve the match between B-factors and computed fluctuations. We also notice differences in relative amplitudes of the experimental and computed atomic fluctuations; these differences exist, however, between the experimental B factors for the two viruses and they could not be interpreted when analyzing the differences between the corresponding structures (Sirohi et al., 2016; Kostyuchenko et al., 2016).

### 4.5. Computing time

**Figure 8:**
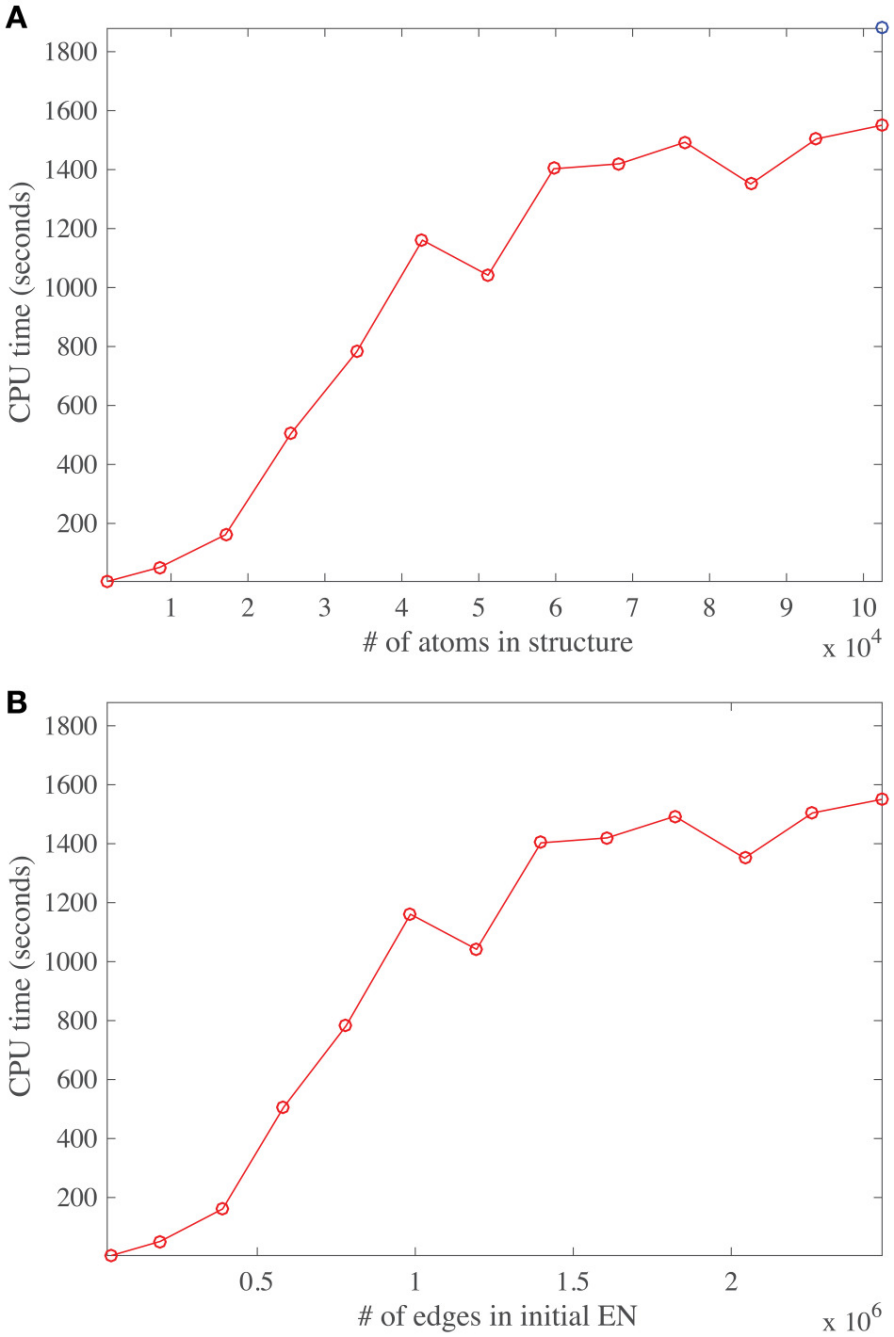
**Running time for DDNMA**. The running time of the normal mode computation is plotted against the initial number of atoms **(A)**, and the initial number of edges in the corresponding elastic network, EN **(B)**. The timings are computed on a single Intel Core I7 processor running at 4.0 GHz with 8 GB of RAM.

The main task performed by DD-NMA when computing the normal modes of an elastic network is the diagonalization of the Hessian. For large systems, it is not feasible to perform the full diagonalization, both because of its time and memory complexities (both of order *O*(*N*^3^), where *N* is the number of atoms). Instead, only partial diagonalization is performed, with only the eigenvalues with the lowest amplitudes (usually 100) being computed. The method implemented is based on an iterative procedure. As discussed in the Material and Methods section, this procedure is efficient, of order *O*(*Mk*+*Nk*^2^ + *k*^3^), where *M* is the number of non-zero elements in the sparse representation of the Hessian matrix, and *k* the number of eigenvalues that are computed. The first term corresponds to the matrix vector multiplications needed at each iteration, the second term relates to the Gram-Schmidt orthogonalization required to build the Krylov basis, and the last term is the cost of diagonalizing the matrix representing this basis. To test if we observe this expected behavior on real systems, we have experimented with systems of varying size. We have applied DD-NMA on parts of the capsids of DENV, with increasing number of asymmetrical units included, from one to sixty. For all systems, we extracted the Cα atoms, computed an all-atom elastic network with a cutoff of 14Å, and computed the 100 lowest frequency normal modes with DD-NMA. All those experiments were performed on a iMAC Apple computer with a 4.0 GHz Intel Core I7 processor, with 8 GB of memory. The computing times for DD-NMA are plotted against the initial numbers of atoms and edges in the all-atom elastic networks in Figure 8.

The number of non-zero elements in the Hessian matrix is directly proportional to the number of edges in the elastic network and implicitly proportional to the number of atoms in the protein, assuming constant density of atoms. Interestingly, the curves cpu time vs. number of atoms and vs. number of edges show three different regimes. For a relatively small number of atoms (below 20,000), and for a medium number of atoms (between 20,000 and 40,000), the cpu time is found to vary linearly, as expected, but with different slopes. The different slopes come from the relative weights of the two terms *Mk* and *Nk*^2^ in the time complexity. For larger number of atoms, the behavior of the cpu time is found to be more erratic, with a slower rate of increase. We suspect that this behavior is due to cache issue. The time complexity of computing the product of the Hessian with a vector using the sparse representation of the Hessian is more complex than just being proportional to *M*, the number of non-zero elements of the Hessian *H*. Indeed, for very large matrices, it depends on their storage patterns. We have not tried to optimize this storage, which is most likely the reason for the erratic behavior. It does hint to possible improvement in the computation of the normal modes, by first re-ordering the Hessian using for example METIS (Karypis and Kumar, 1999).

We note that it takes approximately 30 min to compute the first hundred normal modes for a molecular system with hundred thousand atoms, on a single core, on a desktop computer. While this is not fast *per se*, it is still manageable. We do note that part of the codes for computing the eigenvalues of the Hessian can be parallelized; we are currently working on such an improvement.

## 5. Summary and conclusions

Understanding the dynamics of viral capsids is of fundamental interest for modeling the key steps of viral life cycles. In this paper, we have described an implementation of normal mode analysis based on elastic network models that enables such analyses. This implementation is based on the known foundations in the domain (Tirion, 1996) and does not deviate significantly from other available implementations (Zheng and Doniach, 2003; Suhre and Sanejouand, 2004; Kruger et al., 2012; Tiwari et al., 2014; Eyal et al., 2015; Frappier et al., 2015). We discuss in details its parametrization, namely the choice of the coarse graining of the molecular system, the choice of the method for computing the elastic network, and the assignment of force constants to the resulting springs, and justify the choices we have implemented. We emphasize the need for efficient and robust algorithms for computing the normal modes of elastic networks, in particular when those networks include a very large number of nodes -in the hundred of thousands-, such as those derived for virus capsids. We have illustrated the application of our method to study the dynamics of the viral capsids of DENV serotype 1 and ZIKV. We have characterized the impact of the packing imposed by the capsids on their E proteins that play essential roles in receptor binding and fusion to the membrane of the host cells. We have identified differences in the atomic fluctuations of these proteins between DENV serotype 1 and ZIKV that are consistent with the structural differences observed using high resolution cryo-EM experimental structures (Kostyuchenko et al., 2016; Sirohi et al., 2016). In the future, we will consider two types of extensions of this first study that relate to the method itself as well as to its specific application to studying DENV and ZIKV.

First, we recognize that the need for a reasonable computational cost, when applying a method such as normal mode analysis to a large molecular system such as a virus capsid, implies that some sort of coarse graining is applied to such a system. Many options exist to reduce the dimensionality of the problem by selecting subsets of atoms, “beads,” to represent the system (Kmiecik et al., 2016). The positions of those beads are either defined by known atoms (usually the Cα), or by fitting to capture the dynamics of the full molecular system (Zhang et al., 2008, 2011; Li et al., 2016). The main difficulty in coarse-graining, however, is to design potential energy functions or force fields that retain the physics of the all-atom explicit solvent system in terms of structure, thermodynamics and dynamics (Riniker et al., 2012). While significant efforts have been made to guarantee that a coarse-grained model and its potential capture the complexity of the all-atom molecular system (Riniker et al., 2012; Saunders and Voth, 2013; Na et al., 2015; Zhang, 2015), we note that much less has been done to generate a true multi-scale representation of this system, i.e., to define a hierarchy of coarse-grained models with a coupling between those models. Our intention is to generate such a hierarchy; for this purpose, we will rely on the concept of renormalization group (RG) that is well known in physics (Wilson, 1975). We have implemented in DD-NMA a beta-version of such a method that performs iterative decimation of an elastic network. We will test this method on viral capsids once we have adapted the code to deal with hundreds of thousands of atoms.

Once the representation of the molecular system is chosen, the elastic network is defined as a set of links, with a link between two residues only if the distance between their Cα atoms is smaller than a given cutoff. As an alternative to this cutoff model, Xia et al. (2014) proposed to use all edges of the Delaunay triangulation of the selected atoms as an alternate elastic network. We believe that the use of Delaunay triangulation to define the ENM extends the range of applicability of NMA to the realm of less globular proteins. We will proceed in this direction and test this alternate definition of ENM to study the dynamics of viruses.

Our analyses of the dynamics of DENV and ZIKV capsids were based on naked, empty shells. There are many opportunities to extend this work. We are interested in generating plausible paths between different conformations of the virus capsids, such as the “breathing" induced by increase of temperature (Fibriansah et al., 2013), and the changes observed during the maturation of the virus. We will develop new methods to find such plausible paths in very large systems such as viral capsids, where “plausible" refers to a path with minimal frustration, also defined as the Minimum Action Path (MAP) (Olender and Elber, 1996; Eastman et al., 2001; Franklin et al., 2007; Vanden-Eijnden and Heymann, 2008; Zhou et al., 2008; Chandrasekaran et al., 2016). Finally, we plan to study the impact of glycolsylation of the E protein and/or antibody binding on the virus capsids onto their dynamical properties.

## Author contributions

YH and FP performed research and analyzed data. MD and PK designed research and analyzed data. PK wrote the manuscript.

### Conflict of interest statement

The authors declare that the research was conducted in the absence of any commercial or financial relationships that could be construed as a potential conflict of interest.
